# Immunotherapy Through the Years

**Published:** 2017-11-01

**Authors:** Jessica Eno

**Affiliations:** The University of Texas MD Anderson Cancer Center, Houston, Texas

Immunotherapy’s use in oncology has emerged in recent years as an important treatment option in many different disease sites, but the concept of immunotherapy has been discussed for centuries. Although the beginning of immunotherapy itself dates back to the 18th century, the principles it is based on originated almost 2,000 years prior. The first concepts of immunotherapy were rooted in the principles of infectious disease dating back to the ancient Greeks, most notably Thucydides, who was the first to write about gaining immunity to a specific disease. After surviving the plague that hit Athens in 430 BC, Thucydides observed that survivors were never inflicted with the disease a second time. With this observation, he theorized that survivors developed a resistance to the plague. This became one of the first documented observations about the immune system and laid the groundwork for further study (Piana, 2015). 

## ORIGIN OF THE IMMUNE SYSTEM AND IMMUNOTHERAPY

Even though the initial concept of the immune system was first observed in ancient Greece, the first immunotherapy trial was not conducted until the 18th century in London (Piana, 2015). At this time, Dr. Charles Maitland began studying the deliberate infection of children with small amounts of smallpox. He had previously heard of this practice in Turkey, which prevented those infected from contracting the fulminant disease in the future. He first began by infecting the children of an English Ambassador in Constantinople with pus from someone with active smallpox. Although the children erupted in a pox, after a few days, the rash completely resolved, leaving no scarring or damage.

Hearing about these results, the Princess of Wales allowed Dr. Maitland to "inoculate" six prisoners with smallpox, in hopes of finding a way to protect her children from the deadly disease in the future. After the prisoners recovered from their infection, they were released from prison, and Dr. Maitland exposed them to people with active smallpox. None of the participants on this "trial" became ill with smallpox after reexposure, and Dr. Maitland continued to inoculate people with smallpox to prevent future infections of the disease (Maitland, 1722). As time went on, the concepts of the immune system and immunotherapy in infectious disease were built upon the ideas of the ancient Greeks and Dr. Maitland, but the use of immunotherapy in oncology would not be explored for many years. 

## IMMUNOTHERAPY ENTERS ONCOLOGY

Although studies continued to be conducted about infectious disease, and the field of immunology continued to grow over the following centuries, it wasn’t until the late 19th century that the use of immunotherapy was explored for cancer care (Zacharski & Sukhatme, 2005). Small-scale attempts to use bacterial infections to induce tumor reduction were studied in the late 19th century, but it was not until Dr. William Coley published his first paper on the subject in 1893 that this phenomenon was introduced on a large scale (Wiemann & Starnes, 1994). In this paper, Dr. Coley detailed his experience with using erysipelas infections, an infection of the upper dermis and lymphatics, to treat unresectable sarcomas (Baddour, 2016; Coley, 1893).

Dr. Coley’s interest started when he encountered a gentleman 7 years after the patient underwent surgery to remove his sarcoma, which was originally deemed too extensive and unresectable. However, shortly after the operation, the gentleman developed erysipelas, and his tumor disappeared. After studying erysipelas, Dr. Coley found both anecdotal accounts of tumors disappearing after erysipelas infections, as well as one paper by Dr. Friedrich Fehleisen, who described five cases of unresectable malignant tumors resolving after being exposed to erysipelas. Dr. Coley then decided to inject erysipelas into the first case of unresectable sarcoma he encountered. Although Dr. Coley’s original injections did not create a durable response, the injection of *Streptococcus erysipelatis* caused the patient to have a true erysipelas infection. By the time the infection cleared 10 days later, the patient’s tumor almost completely resolved, and the patient was still living when Dr. Coley published his paper 2 years later (Coley, 1893).

During Dr. Coley’s career, which spanned more than 40 years, he treated almost 900 unresectable sarcoma patients with *Streptococcus erysipelatis*, achieving greater than a 10% cure rate with his "Coley’s toxin" (Wiemann & Starnes, 1994). Despite these results, Dr. Coley’s work was not widely accepted in the medical community, and the idea of using the immune system to fight cancer fell out of favor until the middle of the 20th century. 

The topic of immunotherapy in oncology was almost completely disregarded by the medical community until arguments of its use resurfaced in the late 1940s. Furthermore, disagreement over whether or not immunotherapy could be used in oncology deepened, when, in 1949, Frank Burnet published his "acquired immunological tolerance theory." This theory asserted that self-identifying lymphocytes were destroyed in the prenatal development of the immune system, and therefore, Mr. Burnet claimed, immunotherapy would be impossible because a person’s immune system would not be able to recognize tumor cells that developed from his or her own body. However, during the same period, other researchers were conducting animal experiments showing that once tumors induced by outside carcinogens were removed, they could potentially be immunized against. In many of these experiments, the induced tumor was removed, and then the animals would later reject an injection of the same tumor cells, leading to the idea that tumors have "tumor-associated antigens" (TAA) that the immune system could potentially recognize (Parish, 2003). 

The debate about immunotherapy’s use in oncology was further complicated in the 1960s, when after initially arguing against the use of immunotherapy, Mr. Burnet developed his "immunosurveillance theory," which helped promote the idea of immunotherapy. This theory proposed that lymphocytes function to search tissues for malignant cells through the identification of TAA. This, combined with the TAA experiments of the 1950s, spurred the search to identify targetable TAAs (Parish, 2003). 

**Figure 1:**
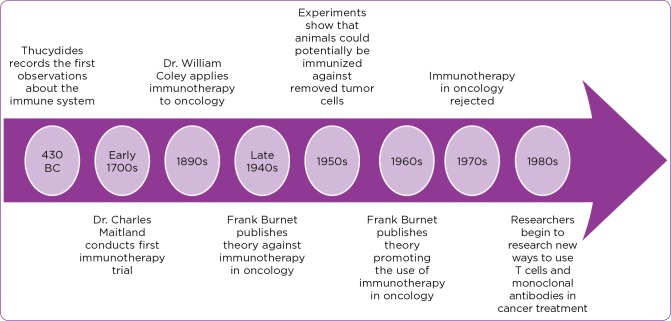
Timeline of immunotherapy in oncology.

The interest in immunotherapy’s role in oncology treatment was short-lived, however, and by the 1970s, Mr. Burnet’s immunosurveillance hypothesis was rejected in favor of his acquired immunological tolerance theory (Parish, 2003). By the 1980s, researchers became cautiously optimistic about the immune system’s ability to detect TAAs, and immunologists began, once again, to search for antibodies that would bind to tumors (Kirkwood et al., 2012). In 1982, Dr. James Allison discovered the T-cell antigen receptor, which laid the groundwork for further identification of how T cells work and how to use them and monoclonal antibodies in cancer treatment (Cavallo, 2014). After decades of debate about the use of immunotherapy in oncology, the discovery of the T-cell antigen receptor tipped the scale in favor of immunotherapy, and immunotherapeutics soon entered development. For a timeline of the emergence of immunotherapy, please see Figure 1.

## EARLY IMMUNOTHERAPEUTICS

With the debate over the ability to use immunotherapy in oncology settled in the 1980s, antitumor cytokines became commercially available to treat multiple disease sites in the late 20th century. The first immunotherapy agent, an antitumor cytokine called interferon-alpha 2 (IFN-a2), was approved by the US Food and Drug Administration (FDA) in 1986, with the expansion and further approval of immunotherapy drugs occurring in the early and mid-1990s.

Interferon-alpha 2 influences the immune system by regulating cytokines and their receptors by both stimulating an innate cell-mediated response as well as creating an adaptive immune response (Brassard, Grace, & Bordens, 2002). Interferon-alpha 2 was initially approved for use in hairy cell leukemia (HCL) after studies showed that IFN-a2 demonstrated a high response rate in patients with progressive HCL (Golomb et al., 1986; Kirkwood et al., 2012). The FDA expanded the approval of IFN-a2 in 1995 to use in the adjuvant treatment of stage IIB/III melanoma.

In 1998, interleukin-2 (IL-2), a T-cell growth factor that aids in immune regulation and T-cell proliferation, became the second antitumor cytokine approved by the FDA when it was approved in the treatment of metastatic melanoma and renal cell carcinoma (Kirkwood et al., 2012). 

In 1990, the FDA approved a new type of immunotherapeutic when it approved the use of intravesicular bacillus Calmette-Guérin (BCG) for noninvasive, stage Tis, Ta, and T1 bladder cancers (Cancer Research Institute, 2016; Redelman-Sidi, Glickman, & Bochner, 2014). It is believed that BCG is taken up by urothelial and tumor cells upon contact. Once internalized by the cells, it is presented to the immune system via antigen-presenting cells (APCs), causing the bladder cells to release cytokines and recruit immune cells to attack any cells containing BCG. Since BCG is also taken up by the tumor cells, the immune system is able to recognize the cells and subsequently attack them (Redelman-Sidi et al., 2014). 

## MODERN IMMUNOTHERAPEUTICS: CHECKPOINT INHIBITORS

After the approval of antitumor cytokines and BCG, researchers continued to look for new ways to use the immune system to fight cancer. Over the past few years, a new class of immunotherapeutics, referred to as checkpoint inhibitors, has emerged as a mainstay in cancer treatment.

Immune checkpoints are the body’s way of protecting healthy cells from damage by the immune system through both the activation and suppression of T cells (Luke & Ott, 2015). These pathways are often activated when proteins on T cells interact with proteins on the surface of other cells in the body. When these proteins interact, they either stimulate T cells to start an immune response against an unfamiliar or ill cell or inhibit T cells from damaging a healthy cell (American Cancer Society, 2015; Dana-Farber Cancer Institute, 2016). However, multiple studies have shown that tumor cells have adapted the ability to express inhibitory receptors seen on healthy cells, which results in decreased function of antigen-specific T cells. Ultimately, this prevents T cells from recognizing and attacking cancer cells (Nguyen & Ohashi, 2014). Checkpoint inhibitors block the interaction of this inhibitory pathway between T cells and tumor cells, allowing the immune system to identify and attack tumor cells (American Cancer Society, 2015).

Two immune checkpoint pathways that are targeted in oncologic immunotherapeutics are the cytotoxic T-lymphocyte–associated antigen 4 (CTLA-4) and the programmed cell death protein 1 (PD-1) pathways, which work at different stages of the immune system. CTLA-4 is activated in the early stages of the immune system, when T cells are mobilized by APCs. Once activated, CTLA-4 is then able to bind to B7 ligands and prevent further T-cell activation (Luke & Ott, 2015). Continued T-cell stimulation by TAAs causes high levels of CTLA-4, which in turn, creates immune cells that can no longer attack the TAA (Tarhini, Lo, & Minor, 2010).

**Figure 2:**
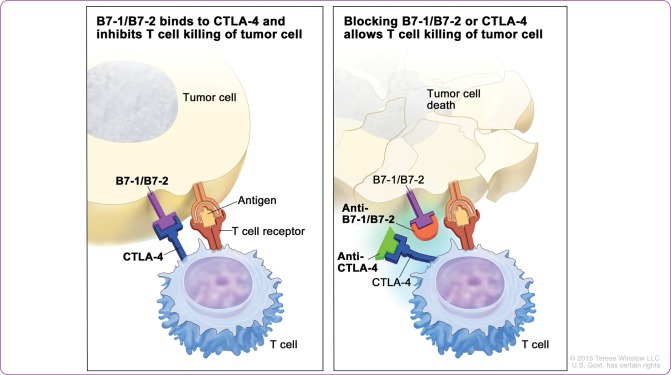
Mechanism of action of CTLA-4 inhibitors. Tumor cells develop B7-1/B7-2 to bind with CTLA- 4 on T cells, which prevents T cells from destroying the tumor cells (left). By blocking the ability of B7-1/B7-2 to bind to CTLA-4 with a CTLA-4 inhibitor, T cells are then able to kill the tumor cells (right). CTLA-4 = cytotoxic T-lymphocyte–associated antigen 4. For the National Cancer Institute © 2015 Terese Winslow LLC, U.S. Govt. has certain rights.

This pathway became the first immune checkpoint target when, in 2011, the FDA approved the anti–CTLA-4 monoclonal antibody ipilimumab (Yervoy) in the treatment of metastatic melanoma (Cancer Research Institute, 2016; FDA, 2015a). Ipilimumab blocks CTLA-4 signaling and its ability to bind to B7 ligands, which heightens T-cell–mediated immunity by perpetuating T-cell activation and restoring T-cell proliferation (Luke & Ott, 2015; Tarhini et al., 2010; Figure 2).

**Figure 3:**
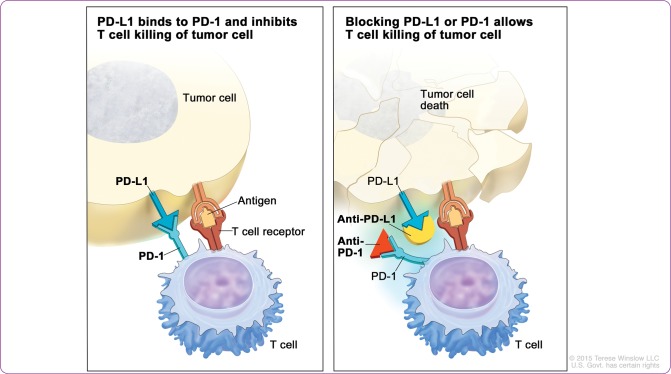
Mechanism of action of PD-1 and PD-L1 inhibitors. Tumor cells develop PD-L1 to bind with PD-1 on T cells, which prevents T cells from destroying the tumor cells (left). By blocking the ability of PD-L1 to bind to PD-1 with a PD-1 or PD-L1 inhibitor, T cells are then able to kill the tumor cells (right). PD-1 = programmed cell death protein 1; PD-L1 = programmed cell death ligand 1. For the National Cancer Institute © 2015 Terese Winslow LLC, U.S. Govt. has certain rights.

The upregulation of the PD-1 pathway occurs later in the immune response, with PD-1 being more prominent on T cells after persistent antigen exposure, such as with chronic infections or tumors (Luke & Ott, 2015). Similar to CTLA-4, tumors can use this pathway to "turn off" T cells and evade destruction. Many different types of tumor cells have evolved to express programmed cell death ligand 1 (PD-L1), which binds with PD-1 on T cells and initiates the inhibitory process (Nguyen & Ohashi, 2014). By blocking PD-1 or PD-L1, the tumor cells are no longer able to initiate the inhibition of T cells, and the immune system is more adequately able to identify tumor cells for destruction (Figure 3). 

In 2014, the FDA approved two PD-1 inhibitors for use in metastatic melanoma: pembrolizumab (Keytruda) and nivolumab (Opdivo; FDA, 2014a, 2014b). Since 2014, the FDA has expanded the use of pembrolizumab and nivolumab for multiple different disease sites, and in 2016, the first PD-L1 inhibitor, atezolizumab (Tecentriq), was approved for use in metastatic urothelial carcinoma (FDA, 2015b–f, 2016a–c). For a complete list of FDA-approved uses of CTLA-4, PD-1, and PD-L1 inhibitors, see the Table on the following page. 

## DISCUSSION

From the first observations of the immune system in ancient Greece to the great debate of the 20th century over the role of immunotherapy in oncology, immunotherapy has emerged from a tumultuous past to become one of the most studied models of cancer care in recent years. The development of BCG, interferon, and IL-2 in the late 20th century began to change the perception of the immune system’s role in oncology, as well as provide new treatment options in multiple different disease sites. However, the rise of checkpoint inhibitors over the past few years has cemented immunotherapy’s place in the care of cancer patients. Furthermore, the development of the first PD-L1 inhibitor has reexamined the PD-1 pathway and may lead to further breakthroughs in immunotherapy in the future. Checkpoint inhibitors have revolutionized treatment modalities in many different types of cancer. As new trials study the efficacy of checkpoint inhibitors in new disease sites, as well as in combination with different agents, new indications for their use will likely be announced.

**Table 1:**
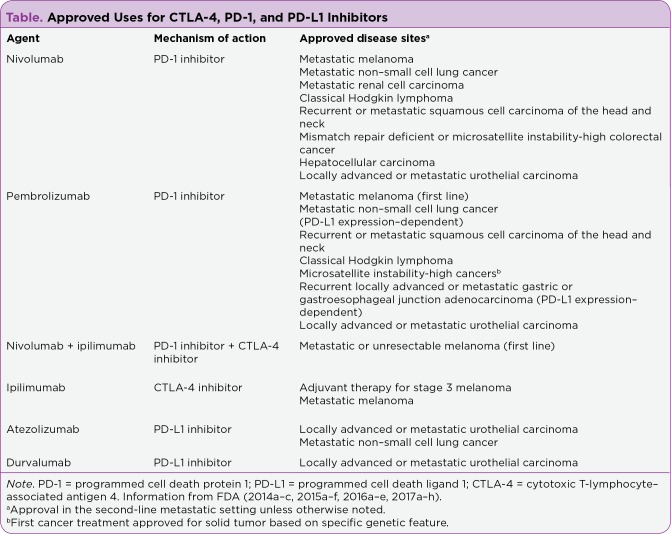
Approved Uses for CTLA-4, PD-1, and PD-L1 inhibitors
